# Impact of Gynecological Interventions on Pelvic Floor Disorders: A Descriptive Analysis of a Case Series in a Hospital-Based Surgical Cohort of 832 Patients

**DOI:** 10.3390/jcm14155244

**Published:** 2025-07-24

**Authors:** Günter Noé, Nele Ziems, Anna Pitsillidi, Ibrahim Alkatout, Dusan Djokovic

**Affiliations:** 1 Department of OB/GYN, University of Witten Herdecke, 58448 Witten, Germany; guenter.noe@uni-wh.de; 2 Department of OB/GYN, Rheinland Klinikum Dormagen, Dr.-Geldmacher-Strasse 20, 41540 Dormagen, Germany; 3 Department of OB/GYN, University of Kiel, 24118 Kiel, Germany; ibrahim.alkatout@uksh.de; 4 Department of Obstetrics and Gynecology, Hospital CUF Descobertas, Rua Mário Botas S/N, 1998-018 Lisbon, Portugal; dusan.d.djokovic@gmail.com; 5 Department of Obstetrics and Gynecology, NOVA Medical School—Faculdade de Ciências Médicas, NOVA University of Lisbon, Campo dos Mártires da Pátria 130, 1169-056 Lisboa, Portugal

**Keywords:** incontinence, pelvic organ prolapse, prospective assessment, risk factors, urogynecological surgery

## Abstract

**Background/Objectives**: Pelvic floor disorders (PFDs) have multifactorial etiology. This makes treatment challenging and often unsatisfactory. This project introduces robust data on risk factors for PFDs and explores opportunities for their prevention, focusing on previous gynecological surgical interventions. **Methods**: We conducted a retrospective analytical cohort study analyzing demographic and clinical data from 832 consecutive patients who underwent pelvic organ prolapse (POP) surgery at a teaching hospital affiliated with the University of Cologne between 2010 and 2019. Patient characteristics—including age, body mass index (BMI), parity, mode of delivery, and symptoms—were collected from medical records. Associations between patient factors and surgical history were assessed using Kendall’s Tau (KT) for correlations and relative risks (RRs) with 95% confidence intervals (CIs) to evaluate the impact of previous hysterectomies and pelvic surgeries on PFD. **Results**: First vaginal delivery and age were the strongest factors associated with PFD. BMI had a smaller impact, and multiple vaginal deliveries did not significantly influence apical (KT 0.037), posterior (KT 0.007), anterior midline (KT 0.015), or lateral defects (KT 0.015). Cesarean section was protective. Subtotal hysterectomy showed no significant association with PFD. Total hysterectomy was strongly associated with posterior defects (RR 4.750, 95% CI: 1.871–12.059) and anterior midline defects (RR 1.645, 95% CI: 0.654–4.139). Recurrent urinary infections were associated with abdominal colposuspension (RR 4.485, 95% CI: 1.12–18.03). Dyspareunia occurred more frequently after vaginal (RR 3.971, 95% CI: 0.78–20.14) and abdominal hysterectomy (RR 1.620, 95% CI: 0.32–8.15). Vaginal hysterectomy was linked to fecal incontinence (RR 5.559, 95% CI: 1.17–26.30), MUI (RR 2.156, 95% CI: 1.09–4.23), and UUI (RR 4.226, 95% CI: 1.82–6.85). **Conclusions:** The factors identified as influencing (PFD) offer a solid foundation for evidence-based patient counseling within our population. Our large dataset confirmed key risk factors, notably childbirth and advancing age. However, the influence of BMI on symptoms and anatomical defects appears to be less significant than previously assumed. Subtotal hysterectomy was not associated with new PFD in our cohort and may represent a viable option when hysterectomy is indicated, though further studies are needed to confirm this potential advantage.

## 1. Introduction

Pelvic floor disorders (PFDs) and the resulting limitations (physical, psychological, and social) affect the lives of many women worldwide [1]. Based on database research from the United States of America, there is a 20% lifetime risk of needing surgical intervention for PFD [2]. Globally, there are great differences in the availability of urogynecological services [3]. It remains a significant challenge worldwide to plan prevention strategies and provide evidence-based patient counseling/treatment. Information on PFDs is commonly either retrospectively taken from state registers or collected by means of questionnaires. Most data are derived from epidemiological studies, which are population-oriented rather than specifically targeting affected patients [4,5]. In addition, the number of women with PFDs but unreported moderate symptoms is unclear even in well-organized health systems. A Dutch study, for example, showed that 47% of hysterectomized women who did not actively seek medical attention for PFDs had relevant clinical complaints [6]. Clinicians and researchers do not reach patients continuously and, therefore, cannot see the full magnitude of the problem and the spectrum of causes. Therewith, this relevant clinical and health economic topic remains somewhat neglected.

To better understand the factors associated with clinically relevant PFD in our female population, we retrospectively analyzed all baseline data of the targeted cohort as part of a follow-up study. We analyzed the obtained information to improve the quality of evidence necessary for adequate clinical counseling and decision-making.

## 2. Materials and Methods

### 2.1. Study Design and Setting

This study is a retrospective analytical cohort analysis conducted at a teaching hospital affiliated with the University of Cologne from 2010 to 2019. Institutional Review Board approval was obtained (approval number 173/19). All participants provided informed consent. The retrospective design with analytical elements justified the calculation of relative risks while recognizing the limitations in inferring causality.

### 2.2. Participants

All patients who underwent POP surgery during the study period and consented to participation were included. Exclusion criteria were incomplete medical records or lack of consent. No sampling was used; the analysis included all eligible cases within the time frame.

### 2.3. Data Collection and Variables

At the initial consultation, demographic and clinical information was collected. Clinical symptoms and relevant previous operations were thoroughly inquired about and meticulously documented by the examining physician. The physical examination was conducted by a specialist in the field of pelvic floor dysfunction. Pelvic floor defects were quantified and staged in each patient using the Pelvic Organ Prolapse Quantification (POP-Q) System [7,8]. A distinction was made between a midline defect and a lateral defect in the case of anterior prolapse (cystocele). Anamnestic data collected from our patients was stored in a password-protected hospital database. Patients’ general data and symptoms were evaluated in relation to current clinical presentation and previous operations. We carried out detailed sub-analyses with regard to previous gynecological surgery in order to evaluate its influence on symptoms and pelvic floor defects.

### 2.4. Statistical Methods

This study was a retrospective cohort study with a descriptive analytical approach. Probabilistic sampling was not used, and no pre-defined hypotheses were tested. Accordingly, no *p*-values or significance testing were applied. The primary outcomes were dichotomous or ordinal, and for these variables, a normal distribution was not assumed.

For continuous variables such as age and body mass index (BMI), the distribution was assessed using the Shapiro–Wilk test and visual inspection of histograms. As these variables did not follow a normal distribution, they are reported as medians with interquartile ranges (IQRs). Categorical variables are expressed as absolute numbers and percentages.

To evaluate factors potentially influencing PFD, association analyses were conducted using Kendall’s Tau (KT) correlation coefficient with corresponding 95% confidence intervals (CIs). KT is a non-parametric measure of ordinal association, ranging from −1 to +1, where values farther from zero indicate stronger relationships. This method was chosen due to its suitability in exploratory studies without inferential testing.

In addition, relative risks (RRs) with 95% CIs were calculated to assess the likelihood of symptoms or anatomical defects associated with previous surgical procedures. Particular attention was given to comparing subgroups of patients with and without hysterectomy, and further distinguishing types of hysterectomy (e.g., subtotal, total, vaginal, abdominal, and laparoscopic). We also analyzed outcomes in patients who underwent combined hysterectomy and colporrhaphy procedures versus those with non-combined surgeries.

### 2.5. Reporting Standards

This study was reported in accordance with STROBE (Strengthening the Reporting of Observational Studies in Epidemiology) guidelines to ensure transparent and comprehensive reporting of observational research.

## 3. Results

Data is based on 832 patients treated surgically in a pelvic floor center (Rheinlandclinic Dormagen; Germany) over 9 years. Their age, body mass index (BMI), obstetrical history, and previous surgeries and symptoms are presented in Table 1. Continuous variables are presented as mean ± SD, while categorical variables are expressed as frequencies and percentages.

### 3.1. Previous Gynecological Surgeries and PFD

In patients previously submitted to abdominal hysterectomy (AH), vaginal hysterectomy (VH), total laparoscopic hysterectomy (TLH), laparoscopic subtotal hysterectomy (LSH), colposuspension (CS), anterior colporrhaphy (AC), posterior colporrhaphy (PC), and tension-free vaginal tape (TVT) or trans-obturator tape (TOT) procedures, determined relative risks for developing stress urinary incontinence (SUI), mixed urinary incontinence (MUI), urge urinary incontinence (UUI), and other symptoms are presented in Table 2.

No significant influence on the severity of the apical defect (n = 832) was found for any previous gynecological procedure. With regard to the posterior defect (n = 209), a protective effect of LSH was evidenced (RR: 0.351, 95% CI: 0.113–1.087). A relationship between VH and the severity of the defect could be observed (RR: 2.318, 95% CI: 1.279–4.199). Regarding laparoscopic CS, there was no significant risk of developing a posterior defect (RR = 0). Regarding abdominal CS, a RR of 3.270 (95% CI: 1.864–5.737) was calculated. After AC (n = 51), there was a significantly increased risk of developing a posterior defect (RR: 3.270, 95% CI: 1.864–5.737). After PC, the recurrence rate was high, resulting in a significant risk increase (RR: 3.246, 95% CI: 1.872–5.630). None of the previous interventions could be found to have a positive or negative effect on the development of midline cystocele.

Table 3 shows PFD risks in hysterectomized vs. non hysterectomized patients. When 73 patients with subtotal hysterectomy vs. 190 women with total hysterectomy were compared, apical defects were similar for both groups. The risk of a posterior defect after total hysterectomy was striking (KT 0.202; RR: 4.750 (95%CI); lo 1.871- hi 12.059). There was also a strong positive tendency for the development of an anterior midline defect after total hysterectomies (RR: 1.645, 95% CI: 0.654–4.139).

### 3.2. Correlations of PFD Symptoms with Age, BMI, and Obstetric History Urinary Incontinence (UI)

1. Urinary incontinence (UI)

Of 362 patients with UI, 265 (73.2%) reported pure SUI [129 (48.7%) grade I, 99 (37.3%) grade II, and 35 (13.2%) grade III; in 2 patients (0.8%) the grade was not recorded; and 58 (16%) patients presented with MUI while 39 (11%) reported UUI.

The age of patients with UI ranged between 32 and 92 years. Patients with SUI were on average 4.03 years younger than patients without UI [mean age ± standard deviation (SD) = 59.03 ± 0.91 years]; those with MUI were 1.49 years older (mean age ± SD = 64.55 ± 1.66 years), and women with UUI were 6.05 years older (mean age ± SD = 69.11 ± 1.97).

BMI ranged from 18.59 to 43.23 kg/m^2^, being on average 0.88 kg/m^2^ higher in women with UI than in those without UI (mean ± SD = 0.36 kg/m^2^). There was a positive correlation between an increase in the mean BMI and an increase in the degree of SUI (grade I: mean BMI = 26.29 kg/m^2^; grade II: 27.62 kg/m^2^; grade III: 29.11 kg/m^2^). In comparison with women without UI, those with MUI and UUI also had a higher average BMI (MUI: +2.20 kg/m^2^, SD = 0.65 kg/m^2^; UUI: +2.37 kg/m^2^, SD = 0.77 kg/m^2^).

There was no statistically significant association between the types of UI and the obstetrical history of cesarian delivery/number of cesarean sections (SUI: KT 0.02, 95% CI −0.019 to 0.058; MUI: KT 0.004, 95% CI −0.018 to 0.026; UUI: KT −0.005, 95% CI −0.020 to 0.009]. However, 80% of patients with a history of a cesarian section also had a vaginal delivery.

2. Repeated urinary tract infections (RUTIs)

RUTIs were reported by 32 patients who were, on average, 6.24 years older than women without RUTIs (SD = ±2.207 years). No significant association was observed between RUTIs and patient BMI or obstetrical history (spontaneous births: KT 0.003, 95% CI −0.029 to 0.036; cesarian section: KT 0.003, 95% CI −0.013 to 0.019).

3. Pain, discomfort, and bulging.

Age, BMI, previous vaginal deliveries, and cesarean sections were not found to be in statistically significant association with these symptoms, reported by 738 patients (mean age ± SD: 62.04 ± 1.518 vs. 62.24 ± 1.518 in women with vs. without pain; mean BMI ± SD = 26.32 ± 4.554 vs. 26.23 ± 4.544; spontaneous births: KT 0.002, 95% CI −0.019 to 0.023; cesarian section: KT 0.002, 95% CI −0.024 to 0.069).

4. Residual urine

Significant residual urine (objectively confirmed by ultrasound: >100mL after micturition) was found in 106 (12.7%) patients. The patients were on average 5.626 years older than those without residual urine (mean age ± SD = 67.11 ± 1.271). A low positive correlation between residual urine and the American Society of Anesthesiologists (ASA) physical status class was observed (KT 0.069, 95% CI 0.024 to 0.114). There was no significant influence of patient obstetric history (KT 0.069, 95% CI −0.024 to 0.114).

5. Fecal incontinence and constipation

Both fecal incontinence reported by 16 (1.9%) patients and constipation reported by 60 (7.2%) patients were found to be only significantly correlated with patient age. In comparison with women without these symptoms (mean age ± SD = 63.86 ± 5.64), patients with fecal incontinence were on average 6.64 years older, while those with chronic constipation were 1.06 years younger.

6. Dyspareunia

This symptom was reported by 22 (2.7%) women. Patients with dyspareunia were on average 13.074 years younger than those without dyspareunia (mean age ± SD = 49.50 ± 3.82 vs. 62.57 ± 2.62). There was a negative association between dyspareunia and the number of spontaneous births (KT −0.022, 95% CI −0.044 to −0.001).

### 3.3. Correlations of POP Defects with Age, BMI, and Obstetric History

1. Apical defects

Apical defects at POP-Q stage I, II, III, and IV were observed in 43 (5.2%), 503 (60.46%) n = 206 (24.76%), and 36 (4.33%) patients, respectively. There was a positive correlation between increasing patient age and defect severity (Table 4) but not between increasing defect severity and increasing BMI (Table 4). The number of vaginal deliveries (KT 0.037, 95% CI −0.014 to 0.089) and the number of cesarean sections (KT −0.024, 95% CI −0.051 to 0.003) had no influence on the severity of level 1 defects.

2. Posterior defects

Posterior defects at POP-Q stage I, II, III, and IV were evidenced in 203 (24.4%), 298 (35.8%), 54 (6.5%), and 5 (0.6%) patients, respectively. There was a positive correlation between increasing age and severity of the rectocele (Table 4). There was no correlation for BMI (Table 4) as well as for the number of spontaneous deliveries (KT 0.007, 95% CI −0.054 to 0.068) and cesarean sections (KT −0.009, 95% CI −0.039 to 0.021).

3. Anterior midline defects

Anterior midline defects (cystocele) at POP-Q stage I, II, III, and IV were clinically diagnosed in 88 (10.6%), 183 (22.0%), 155 (18.7%), and 20 (2.4%) patients, respectively. There was a positive relationship between defect severity and increasing age (Table 4). A negative correlation was found between defect grading and BMI (Table 4). There was no significant association between the number of spontaneous births (KT 0.015, 95% CI −0.059 to 0.089) and the number of cesarean sections (KT −0.012, 95% CI −0.041 to 0.016).

4. Lateral defects

Lateral defects at POP-Q stage I, II, III, IV, and 0 were evidenced in 85 (10.2%), 283 (34%), 89 (10.7%), 3 (0.36%), and 372 (44.7%) patients, respectively. For the lateral defect, no correlation between age and increase in the defect was found (Table 4). For BMI, there was a positive correlation with stage I to III, while there was insufficient data for stage IV (Table 4). There was no correlation regarding the number of spontaneous births (KT 0.015, 95% CI −0.051 to 0.080) or the number of cesarean sections (KT 0.014, 95% CI −0.024 to 0.052).

In Table 5, correlations between the anamnestic data and symptoms as well as pelvic floor defects observed in our patients are presented.

## 4. Discussion

PFDs are related with a significant increase in global health costs and challenges. Therewith data on clear risk factors but even more on preventive strategies get utmost importance. There is a wealth of epidemiological data on risk factors for developing PFDs. When interpreting these data, it is crucial to recognize the multifactorial nature of the disease, which makes it necessary to evaluate individual risk factors in their broader context in order to develop individualized treatment with low risk and the best possible success rate.

We prospectively assessed a large German citizen population and analyzed correlations between patients’ anamnestic data, symptoms, and objective PFD signs. As expected, vaginal birth has been clearly identified as the strongest PFD risk/ethological factor in many studies [9,10,11,12]. Handa et al. have identified vaginal surgical interventions to be a strong risk factor as well [13]. According to a metanalysis by Hagen-Franzen et al., the risk for long-term UI seems to be very high when it occurs during pregnancy [14]. Our data support the importance of the influence of vaginal delivery on the development of PFDs as only 3.2% of affected patients had not given birth. While the first vaginal birth seems to be decisive, we did not find that increased parity (i.e., the number of vaginal deliveries > 1) further influenced the symptoms and the different pelvic defects. This was also indicated by Quiroz et al. in a study with 290 patients [15]. In addition, our patients with several vaginal deliveries suffered significantly less often from dyspareunia.

DeLancey et al. point to the anatomical load on the pelvic floor structures, recommending research into the effects of the avoidance of manual rotation, slow birth guidance, and possible earlier induction of labor, and suggesting that women giving birth should be instructed not to push against tense pelvic floor muscles [10]. Other researchers addressed the impact of cesarean section on pelvic floor protection. Various authors have attempted to assess these issues in the form of literature reviews [16], ultimately without success due to large research variations in terms of time, characteristics of the populations studied, and overall heterogeneity of the data. Nevertheless, there are protective measures that are obvious, such as the renunciation of forceps deliveries whenever possible. Memon et al. [11] estimate that instrumental delivery increases the risk of pelvic muscle injury and nerve stretching by two to three times. In addition to POP, incontinence rates also appear to be significantly lower in women who have given birth exclusively via cesarean section. Among our patients with PFDs, only 1.6% delivered exclusively by cesarean section. Therefore, the underrepresentation of exclusive cesarean sections in our PFD–affected population may indirectly lead to the conclusion that cesarean sections have a protective effect. Still, the discussion on cesarean section and vaginal delivery goes far beyond the risk of POP and addresses medical as well as social and economic aspects.

Vergelt et al. demonstrated in a systematic review that, in addition to parity and vaginal delivery, patient age and BMI are risk factors for POP [17], while preoperative prolapse stage is a risk factor for POP recurrence [18]. While it seems undisputed that vaginal birth is the decisive factor in incontinence and descensus, age also makes a significant contribution [19], which is indicated by our results as well. On the other hand, our data do not prove that BMI is in general a significant contributing factor in the study population with POP. Only the risk for SUI tended to be increased. A review and metanalysis by Zanebe et al. also indicated the low impact of BMI [20].

Both the American Association of Gynecologic Laparoscopists (AAGL) and the International Society for Gynecologic Endoscopy (ISGE) recommend that VH should be considered the approach of first choice for benign pathology [21,22,23]. Regarding post-hysterectomy POP development, there is increasing evidence that total hysterectomies and especially VH have disadvantages for patients. A Danish registry study with over 88.000 patients showed a 40% increased risk of patients suffering from descensus if they were hysterectomized. For the posterior compartment, the risk was increased by 2.2 times [24]. A British study over decades showed even higher risks [4]. We performed an inter-hysterectomy analysis as well as a comparison with non-hysterectomized patients. Our data did not confirm that VH is as a risk factor for SUI alone but MUI and UUI were 2.2 and 4.2 times more often associated with VH, respectively. Fecal incontinence also occurred exclusively after VH. If we look at the different approaches of hysterectomy in relation to the non-hysterectomized patients, it is noticeable that LSH is almost equivalent to patients without hysterectomy. VH in particular increased the risk of a posterior wall defect, and AH slightly lowered it. These results confirm previous observations. Subtotal hysterectomy is strongly underrepresented overall due to fear of cervical carcinoma but should be increasingly considered if there is no contraindication, according to the data available. Ultimately, the influence of benign hysterectomy on the overall incidence of cervical cancer is low and no longer relevant in countries with surveillance. Therefore, the general statement that vaginal hysterectomy is generally preferable should also be reconsidered. In addition, the recommendations of different associations may need to be clarified, for example, on the availability of laparoscopy. This applies in particular to those regions of the world where MIS surgery is generally available and widely accessible. Complementary evaluation of a large number of patients in other populations, with uniform data documentation and physical examination, may allow a clearer understanding of the factors influencing PFD.

It is important to acknowledge that this study’s retrospective, single-center design introduces limitations. Selection bias may affect the findings, and reliance on medical records could omit relevant variables. Moreover, the retrospective nature prevents establishing causality. These factors limit the generalizability of our results. Future prospective, multicenter studies with standardized data collection are needed to validate and expand upon these observations.

## 5. Conclusions

To sum up, in our patients, complete hysterectomies, especially VH, significantly increased the risk of posterior wall defects, UUI, and MUI. Subtotal hysterectomy shows no impact on pelvic floor disorder and can be considered a preventive strategy for POP in case of necessary hysterectomy without contraindication to preserve the cervix. The first vaginal delivery had the greatest impact besides age on PFD development. The effect of BMI was much smaller than often assumed. Subtotal hysterectomy had no effect on UI/POP. These insights emphasize the importance of individualized patient counseling and careful consideration of surgical approaches when managing and preventing PFD. Integrating this knowledge into clinical decision-making can help optimize treatment outcomes and minimize risks.

## Figures and Tables

**Table 1:** Demographic and clinical characterization of patients included in the study.

**General data:**	
Median age (IQR), [years]	63 (52–72)
Median body mass index (IQR), [kg/m^2^]	25.7 (23.2–29.0)
**Obstetrical history:**	
One or more vaginal deliveries, n (%)	803 (96.9)
Cesarean section(s), n (%)	65 (7.8)
Both cesarian and vaginal deliveries, n (%)	52 (6.2)
Cesarean sections only, n (%)	13 (1.6)
**Personal surgical history**	
At least one previous surgery, n (%)	322 (38.7)
Multiple previous operations, n (%)	122 (38)
Laparoscopic subtotal hysterectomy, n (%)	73 (22.7)
Abdominal hysterectomy, n (%)	63 (19.6)
Vaginal hysterectomy, n (%)	122 (37.9)
Total laparoscopic hysterectomy, n (%)	5 (1.6)
Colposuspension, n (%)	26 (8.1)
Anterior colporrhaphy, n (%)	72 (22.4)
Posterior colporrhaphy, n (%)	59 (18.3)
Tension-free vaginal tape or trans-obturator tape procedures, n (%)	15 (4.7)
**Symptoms**	
Urinary incontinence, n (%)	362 (43.5)
Recurrent urinary tract infections, n (%)	32 (3.9)
Pain/discomfort and bulging, n (%)	738 (88.7)
Residual urine, n (%)	106 (12.7)
Fecal incontinence, n (%)	16 (1.9)
Chronic constipation, n (%)	60 (7.2)
Dyspareunia, n (%)	22 (2.6)

**Table 2:** Previous surgery influence on pelvic floor symptoms (risk ratio).

Surgery (Number of patients, percentage in the total population)	LSH (73–8.7%)	TLH (5–0.6%)	VH (122–14.6%)	AH (63–7.6%)	CS ab/lsc. (13/13–1.6%)	AC (72–8.7%)	PC (59–7%)	TVT/TOT (15–1.8%)
**Stress urinary incontinence,** **risk ratio**	1.07 (0.73–1.45)	1.15 (0.39–3.39)	0.77 (0.55–1.092)	1.207 (0.836–1.743)	1.31/0.95 (0.67–2.66/0.42–2.14)	0.69 (0.44–1.04)	0.53 (0.30–0.93)	1.34 (0.73–2.46)
**Mixed urinary incontinence,** **risk ratio**	0.83 (0.35–1.95)	-	2.16 (1.09–4.23)	0.684 (0.249–1.878)	0.89 (1.93–5.33/1.68 (0.45– 6.45)	1.14 (0.51–2.53)	1.49 (0.67–3.27)	2.4062 (0.84–6.90)
**Urge urinary incontinence,** **risk ratio**	-	-	4.23 (1.82–6.85)	1.05 (0.36–2.98)	1.29 (0.19–8.74)/-lsc.	-	0.81 (0.25–2.66)	5.09 (1.99–12.97)
**Repetitive urinary infections,** **risk ratio**	0.67 (0.15–2.93)	-	0.99 (0.33–2.93)	2.53 (0.86–7.46)	4.49 (1.12–18.03)/0	0.28 (0.04–2.10)	0.35 (0.05–2.65)	-
**Residual urin,** **Risk-ratio**	0.59 (0.24–1.46)	-	1.01 (0.54–1.89)	1.78 (0.93–3.42)	2.242 (0.80–6.29)/ 1.42(0.39–5.35)	1.11 (0.55–2.25)	1.41 (0.70–2.83)	0.60 (0.09–4.07)
**Fecal incontinence,** **risk ratio**	-	No data	5.56 (1.17–26.30)	0.50 (0.07–3.97)	-/3.08 (0.42–22.72)	4.17 (1.15–15.15)	3.38 (0.94–12.19)	-
**Dyspareunia,** **risk ratio**	-	-	3.97 (0.78–20.14)	1.62 (0.32– 8.15)	-	1.65 (0.34–8.49)	2.50 (0.34–8.49)	-

-: no patient with the symptom, 95% CI: 95% confidence interval; AC: anterior colporrhaphy; AH: abdominal hysterectomy; CS ab/lap: colposuspension abdominal/laparoscopic; LSH: laparoscopic subtotal hysterectomy; PC: posterior colporrhaphy; RR: relative risk; TLH: total laparoscopic hysterectomy; TOT: trans-obturator tape procedure; TVT: tension-free tape procedure; VH: vaginal hysterectomy; Gray fields: statistically significant risk.

**Table 3:** Incontinence and pelvic organ prolapse risks in hysterectomized vs. non hysterectomized patients.

Symptom/Defect	LSH vs. No Hysterectomy	VH vs. No Hysterectomy	AH vs. No Hysterectomy	TLH vs. No Hysterectomy
**Stress urinary continence,** **Kendall’s Tau/RR**	0.003/1.033	-/0.835	0.145/1.145	0.124/1.124
**Urge urinary incontinence,** **Kendall’s Tau/RR**	0.001/1.0	-/3.530	0.935/1.935	-
**Apical defect,** **Kendall’s Tau/RR**	0.0004/1.032	0.013/1.031	0.016/1.238	-
**Posterior defect,** **Kendall’s Tau/RR**	0.006/1.167	0.210/4.750	−0.059/2.789	-
**Anterior midline defect,** **Kendall’s Tau/RR**	0.052/0.837	0.022/0.997	0.044/0.833	-
**Lateral defect,** **Kendall’s Tau/RR**	−0.012/0.775	0.002/0.671	0.012/1.300	-

-: no patient with the symptom, 95% CI: 95% confidence interval; AH: abdominal hysterectomy; LSH: laparoscopic subtotal hysterectomy; RR: relative risk; TLH: total laparoscopic hysterectomy; VH: vaginal hysterectomy; Gray fields: statistically significant risk.

**Table 4:** Patient age, BMI, and POP severity.

Prolapse and Grade	Mean Age ± SD [Years]	Mean BMI ± SD [kg/m^2^]
**Apical**		
I	59.05 ± 9.45	27.63 ± 5.39
II	60.28 ± 12.14	26.36 ± 4.51
III	64.37 ± 11.98	26.16 ± 4.27
IV	69.39 ± 12.66	27.13 ± 4.10
**Posterior**		
I	60.80 ± 12.14	26.87 ± 4.61
II	62.57 ± 11.46	26.00 ± 4.23
III	65.59 ± 10.69	27.13 ± 4.83
IV	69.80 ± 7.46	24.97 ± 3.43
**Anterior midline**		
I	59.51 ± 12.119	27.63 ± 4.54
II	65.57 ± 10.686	26.60 ± 4.53
III	67.11 ± 8.856	26.32 ± 3.67
IV	73.70 ± 9.772	25.17 ± 4.07
**Lateral**		
I	60.77 ± 12.46	26.16 ± 4.86
II	57.17 ± 11.51	26.26 ± 4.63
III	58.79 ± 12.44	26.88 ± 5.19
IV	64.33 ± 14.22	23.63 ± 3.20

**Table 5:** Summary of the influence of patient age, BMI, and obstetrical history on PFD (deviation of Kendall’s Tau from 1).

	Age	BMI	Vaginal Delivery	Cesarean Section
**Stress urinary incontinence**	+++ positive	+ positive	-	-
**Mixed urinary incontinence**	++ positive	+ positive	-	-
**Urge urinary incontinence**	+++ positive	+ positive	-	-
**Repetitive urinary infections**	+++ positive	-	-	-
**Pain, discomfort and bulging**	-	-	-	-
**Residual urine**	+++ positive	-	-	-
**Fecal incontinence**	+++ positive	-	-	-
**constipation**	++ positive	-	-	-
**Dyspareunia**	+++ negative	-	+ negative	-
**Apical defect**	++ positive	-	-	-
**Posterior defect**	++ positive	-	-	-
**Midline cystocele**	++ positive	+ negative	-	-
**Lateral defect**	-	+ positive	-	-

-: no correlation; +: influence evident (negative/positive); ++: clear correlation (negative/positive); +++: strong correlation (negative/positive).

## Data Availability

The original contributions presented in this study are included in the article. Further inquiries can be directed to the corresponding author(s).

## Abbreviations

The following abbreviations are used in this manuscript:

AAGL	American Association of Gynecologic Laparoscopists
AC	Anterior colporrhaphy
AH	Abdominal hysterectomy
ASA	American Society of Anesthesiologists
BMI	Body mass index
CI	Confidence interval
CS	Colposuspension
ISGE	International Society for Gynecologic Endoscopy
KT	Kendall’s Tau coefficient
LSH	Laparoscopic subtotal hysterectomy
MUI	Mixed urinary incontinence
PC	Posterior colporrhaphy
PFD	Pelvic floor disorder
POP	Pelvic organ prolapse
POP-Q	Pelvic Organ Prolapse Quantification
RR	Relative risk
RUTIs	Repeated urinary tract infections
SD	Standard deviation
SUI	Stress urinary incontinence
TLH	Total laparoscopic hysterectomy
TOT	Trans-obturator tape
TVT	Tension-free vaginal tape
UUI	Urge urinary incontinence
VH	Vaginal hysterectomy
